# Recent Incarceration and Minoritized Racial Status as Barriers to the Effectiveness of Recovery Management Checkups

**DOI:** 10.21203/rs.3.rs-5448616/v1

**Published:** 2024-12-18

**Authors:** Jeffrey Kahn, M. Kate Hart, Dennis P. Watson, Caroline B. Allen, Ryan R. Singh, Christine E. Grella, Michael L. Dennis

**Affiliations:** Illinois State University; Chestnut Health Systems; Chestnut Health Systems; Chestnut Health Systems; Chestnut Health Systems; Chestnut Health Systems; Chestnut Health Systems

**Keywords:** Recovery management checkups, substance use, returning citizens, minoritized status

## Abstract

**Background:**

Recovery management checkups done in primary care settings (RMC-PCs) can be an effective intervention to link individuals with substance use disorders (SUD) to treatment and help them stay engaged with treatment. There is reason to question, however, whether RMC-PCs are as effective for those who have been recently incarcerated or for those holding a minoritized, racial identity.

**Methods:**

We examined data from a randomized controlled trial of RMC-PCs compared to a control condition (*N* = 266).

**Results:**

Multilevel analyses of 4-wave data (3, 6, 9, and 12 months after baseline) indicated that RMC-PCs were more effective than the control condition, especially early in the 12-month study period. The relative effectiveness of RMC-PCs was stronger, however, for participants with fewer days in jail just prior to baseline as well as for White (versus non-White) participants.

**Conclusions:**

These findings suggest the utility of examining potential mediators of these moderated effects in future research as well as tailoring SUD interventions to better meet the needs of these populations.

## BACKGROUND

The number of people currently incarcerated in the United States is substantial. Over 1 million are currently held in U.S. prisons [1] and, across a 12-month period during 2022–2023, local jails reported 7.6 million admissions [2]. Moreover, the prevalence of substance use disorders (SUD) among the incarcerated population is alarmingly high [3, 4]. This heightened prevalence is associated with multiple problems. For example, substance use is associated with increased recidivism [5] and suicidality [6], making the treatment of SUD to those who are incarcerated a critical need.

In fact, the need for treatment extends beyond those presently in jails and prisons to those who have recently returned to the community (i.e., *returning citizens*). However, barriers to treatment exist among returning citizens, including lack of motivation to enter treatment, concerns about privacy, and concerns about access to treatment [7]. In addition, many individuals lose Medicaid coverage while incarcerated or otherwise lack funds to pay for treatment [8]. Access to treatment following release to the community is further hindered by limited interorganizational relationships between carceral facilities and community providers [9], logistical challenges, and resistance to referring individuals to SUD treatment from community corrections [10].

Returning citizens are also at heightened overdose risk, especially in the days immediately following release [11–13]. While many factors may contribute to this increased risk, a history of incarceration, no matter how recent, is associated with decreased harm-reduction behaviors regarding substance use. For example, Victor et al. (2024) examined the harm-reduction behaviors of individuals with a past-year opioid use disorder who expressed interest in treatment [14]. As compared to those with no incarceration histories, they found those with incarceration histories were less likely to report engaging in a variety of harm-reduction behaviors (e.g., having naloxone available, using a trusted supplier). Returning citizens may also face housing challenges that interfere with recovery [15]. These factors add to the concern that interventions for SUD may be less effective for returning citizens.

Recent incarceration is not the only potential barrier to SUD treatment—so is one’s race or ethnicity. Indeed, there are numerous racial and ethnic discrepancies related to incarceration, substance use, and treatment. For example, Black individuals are overrepresented among those who are incarcerated; at the same time, the rate of incarceration for drug offenses among the Black population has grown at a faster rate than for other populations [16]. Racial disparities also extend to SUD treatment among incarcerated individuals. Wells et al. (2001) reported that among those incarcerated, White individuals were more likely than Latino/a or Black individuals to receive alcohol or drug treatment [17]. Similarly, Nowotny’s (2015) analysis of 5,180 individuals who were incarcerated across 286 correctional facilities revealed that Latino/a people were significantly less likely than White people to receive SUD treatment [18]. Even after incarceration, there are reasons to consider race/ethnicity as a contextual factor in SUD treatment. Research has suggested that Black and Hispanic populations are less likely to complete SUD treatment across all settings compared to White populations [19]. Similarly, Victor et al. (2024) found that White participants were significantly more likely than Black participants to use fentanyl test strips as a harm reduction measure to test for drug adulteration [14]. Although research in jails has not found racial/ethnic discrepancies in treatment or use of services [20], substantial research suggests incarceration history and race/ethnicity are important contextual variables when considering substance use and participation in SUD treatment. Given this, we sought to examine the roles of these contextual variables on the efficacy of an SUD treatment linkage intervention.

### Recovery Management Checkups (RMCs)

One potentially valuable approach to increasing access to SUD treatment is the use of Recovery Management Checkups (RMCs). RMCs are interventions that target individuals in the community with SUD who face barriers to entering or staying in treatment [21]. RMCs are predicated on a chronic disease model in which individuals with SUD tend to transition through cycles of use, treatment, incarceration, and recovery [22]; this cyclical pattern has been demonstrated in numerous prior longitudinal studies [22, 23]. RMCs, therefore, involve regular checkups with the individual to ensure linkage to treatment and to support their treatment retention and recovery over time [22].

Although approaches such as Screening, Brief Intervention, and Referral to Treatment (SBIRT) also provide referrals as needed, evidence for SBIRT’s effectiveness as a stand-alone approach is weak [24]. The RMC approach is promising because it links the individual to treatment, maintains engagement with the individual, and supports their treatment retention [22]. Ongoing monitoring is part of RMCs, which helps ensure treatment can be provided if a relapse occurs. Moreover, RMCs have been successfully implemented in primary care settings (RMC-PC) [25], such as Federally Qualified Health Centers (FQHCs). Involving FQHCs with RMC is particularly important as FQHCs serve large numbers of patients who may be at risk for SUD given these patients’ health disparities, marginalized racial or ethnic identity (e.g., Black, Hispanic), and barriers to receiving services [25].

A substantial body of research supports the efficacy of quarterly RMCs in terms of outcomes such as return to treatment, reduced time to treatment entry, and more days of treatment received over time [21, 22, 26–29]. Specifically, days of treatment seem to mediate the association between receipt of RMC (versus SBIRT only) and days of alcohol and drug use [21, 25]. Given this evidence, RMCs would be an ideal intervention to examine when evaluating the effect of recent incarceration and racial minoritized status on the efficacy of an SUD linkage intervention. This is especially true because individuals who have recently been incarcerated often face many challenges when reintegrating into the community; thus, such an investigation could potentially address barriers these individuals face to accessing SUD treatment in the community. When comparing RMC to the control group among women offenders recently released from jail, Scott and Dennis found RMC more effective on returning to and participating in treatment within the first 90 days post-release [30]. An important note is that 83% of women in this study identified as African American; this begs the question of whether those who identify as White would experience similar outcomes [30].

### The Present Study

The purpose of this study was to examine the role of prior incarceration and racial minoritized status on the effectiveness of RMC for patients with SUD in primary care settings (RMC-PC). The research questions were whether (a) time in jail before study enrollment and/or (b) status as a racial minoritized participant interfered with the effectiveness of RMCs compared to a control condition (SBIRT only). Effectiveness in this study was defined as use of alcohol or other drugs [21, 25]. Whereas there is reason to believe SUD interventions, in general, would be less effective for those who were recently incarcerated and/or identify as non-White, RMC-PC seems particularly well-suited to address the unique challenges associated with these contextual variables. Given these competing perspectives, we did not make specific hypotheses concerning the relative effectiveness of RMC-PC for these populations. Our study was, therefore, exploratory in this regard.

## METHODS

### Participants and Procedure

Data for this study came from a randomized controlled trial of RMC-PC conducted from 2017 to 2020 [21]. Participants (*N* = 266) were adult patients in one of four Federally Qualified Health Centers (FQHCs) in Chicago, IL. The majority identified as male (65%) and African American (81%); reported being age 50 or over (54%); and stated they held at least a high-school degree or equivalent (58%). There were no significant differences in participant characteristics by study condition; see Scott et al. (2023) for data on participant characteristics [21].

All participants received SBIRT—a standard practice for these FQHCs—before study enrollment and randomization. SBIRT involves screening to determine whether alcohol or drug treatment is needed along with brief interventions and referrals administered by clinic staff (i.e., nurses, health educators). Patients who received SBIRT at these sites were deemed eligible for the study provided they met additional inclusion criteria: (a) scoring at a moderate to high level on the Alcohol Use Disorder Identification Test (AUDIT 5) [31] or the Drug Abuse Screening Test (DAST 3+) [32]; (b) receiving a referral for drug or alcohol treatment prior to randomization; and, (c) not currently receiving any alcohol or drug treatment. (Exclusion criteria are described by Scott et al. (2023) [21]). Research staff contacted eligible participants to describe the study in more detail. Those who agreed to participate provided their informed consent and completed the baseline assessment.

At this point, participants were randomized with about half of the participants (*n* = 132) randomly assigned to not receive any intervention beyond the SBIRT; this was the study’s control condition. The other half (*n* = 134) were randomly assigned to receive RMC-PC as well as the SBIRT. The linkage managers who deliver the RMC-PC intervention contacted participants two to three times each week during the first two weeks of study enrollment to support treatment retention (for those currently in treatment) or encourage and link participants to treatment if warranted. Using results from the baseline assessment, linkage managers provided individualized feedback to participants regarding their alcohol and/or drug use, discussed the participants’ motivation to abstain from substances, and any potential barriers to receiving treatment. Using motivational interviewing, the RMC-PC linkage managers bolstered participants’ commitment to change, particularly for those who continued to use substances. Over the 12-month study period and depending on the individuals’ current status, linkage managers provided quarterly check-ups, re-engaged individuals in SUD treatment as needed, supported participants’ treatment retention, and provided support for maintaining recovery. Additional details about these interventions are provided by Scott et al. (2023) [21].

### Measures

Assessments occurred prior to randomization at baseline and again 3, 6, 9, and 12 months after randomization. The 1-hour interviews were conducted either in person or by phone and included the GAIN-Q3 [33, 34], a 30-minute standardized interview covering problem areas (e.g., physical health, stressors, psychiatric symptoms) as well as substance use, SUD-related problems, and SUD treatment.

The outcome variable was past 90-day use of alcohol or other drugs, specifically, the number of days any drug was used. Sixteen GAIN-Q3 questions assessed the use of alcohol, marijuana, crack/other cocaine, heroin/other opioids, hallucinogens, methamphetamine, and the misuse of prescription medications. The prompt for these questions was, “During the past 90 days, on how many days have you used…?” [34]. This outcome variable was computed as the maximum number of days of use for any assessed substance; thus, scores reflected a conservative estimate of the number of days (from 0 to 90) in which alcohol or any other substance was used. Because past 90-day use was assessed at five time points (including baseline), we were able to use prior 90-day use as a control when predicting subsequent 90-day use.

Days in jail during the 90 days prior to intake was a continuous variable between 0 and 90. This variable was positively skewed (skewness = 7.05), with only 18% of the participants serving jail time prior to intake. Despite this, jail days was preserved as a continuous variable to retain power to detect effects across different number of days in jail. Minoritized status was coded as 1 = non-White and 0 = White. Ninety-one percent of participants identified as non-White.

## RESULTS

### Preliminary Analyses

Given the underlying model that RMC-PC would reduce substance use via days of treatment, we examined whether participants in the RMC-PC condition reported having had more days of treatment throughout the 12-month study period than participants in the control condition (see Scott et al. (2023) for more expansive results) [21]. Indeed, this was the case, *t*(257.13) = 2.15, *p* = .03, *d* = 0.26 (see Table 1). There were more treatment days for the RMC-PC condition than for the control condition in each 90-day period, although only the difference in the first 90 days was statistically significant, *t*(259.53) = 2.12, *p* = .04, *d* = 0.26.

We also examined the association between days in jail and racial, minoritized status. Participants identifying as non-White had more jail days (*M* = 1.42, *SD* = 7.05) than participants who identified as White (*M* = 0.16, *SD* = 0.56), *t*(1045.66) = − 5.35, *p* < .001, *d* = − 0.19. Moreover, 19% of non-White participants reported having spent time in jail in the 90 days prior to the study compared to 9% of White participants. Thus, racial minoritized status and jail days were correlated with each other, although we note that the effect size (Cohen’s *d*) of this association is small [35].

### Moderation of Days in Jail on RMC-PC Effectiveness

The first research question concerned examining whether time in jail during the 90 days before baseline related to the effectiveness of RMC-PC. We tested three hierarchically nested multilevel models predicting the number of days in which alcohol or other drugs would be used in the subsequent 90-day period. Analyses were completed using R’s nlme package. The first model was a main-effects model with time (i.e., four 3-month intervals beginning with 0–3 months and ending with 9–12 months), intervention condition (0 = SBIRT only and 1 = SBIRT + RMC-PC), and number of jail days. In addition, the number of days in which alcohol or other drugs (AOD) were used in the prior 90-day period was used as an additional control. Model 2 added two-way interactions among time, treatment condition, and jail days, and Model 3 added the three-way interaction among these three variables. Random effects were specified for the intercept and jail days; a random effect for prior AOD use led to convergence issues, so this random effect was omitted from the model. Sample sizes for these analyses were *N* = 266 participants with *N* = 1051 observations (*M* = 3.95 observations per participant).

Prior to testing the model, the intraclass correlation (ICC) was computed for AOD use. The ICC was .57 which indicates that 57% of the variance in AOD use was between-person. Model 1 revealed that prior 90-day AOD use was positively related to subsequent AOD use, yet no other main effects were significant (see Table 2).

**Figure 1:**
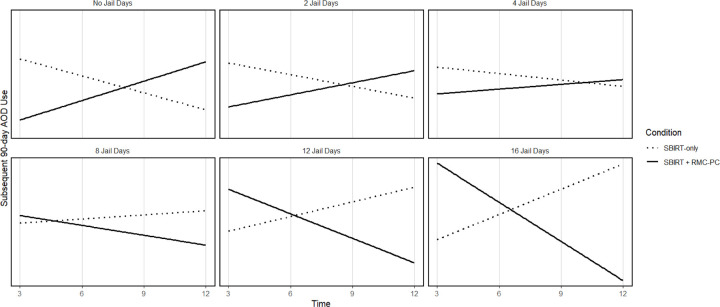
Simple Slopes between Time of the Assessment and Days of AOD Use for the Two Intervention Conditions at Varying Numbers of Jail Days Prior to Intake

Model 2 added two-way interactions, and this model revealed a significant two-way interaction between time and intervention condition. Specifically, whereas those receiving RMC-PC had fewer days of AOD use than those only receiving SBIRT early in the intervention period, this effect decreased over time. In Model 3, there was a significant two-way interaction between condition and days in jail before baseline; this interaction indicated that when compared to SBIRT, the effectiveness of RMC-PC was strongest for those with fewer days in jail. However, this two-way interaction was qualified by a significant three-way interaction among intervention condition, time, and days in jail before study intake. This three-way interaction is displayed in Fig. 1. With no days in jail, the effectiveness of RMC-PC compared to SBIRT-only was apparent early in the intervention period, yet when participants had spent more days in jail before intake, there was decreased effectiveness of RMC-PC during the initial period (e.g., the first 3 months). This pattern of findings suggests that RMC-PC is not as effective during the initial period of the intervention (i.e., just after study enrollment) for those with greater time in jail prior to study enrollment.

### Moderation of Racial Minoritized Status on RMC-PC Effectiveness

Our second research question was to examine racial, minoritized status as a predictor of days of AOD use over the follow-up along with its potential interactive effect involving intervention condition and time. We again tested three hierarchically nested models: (1) main effects only; (2) two-way interactions among time, intervention condition, and minoritized status; and (3) an additional three-way interaction among these variables. As before, we controlled for prior 90-day AOD use and also included prior 90-day AOD use as a random effect (along with random effects for time and the intercept).

There was a strong, positive association between prior 90-day AOD use and subsequent 90-day AOD use in Model 1 (see Table 3). Model 2 also revealed the same two-way interaction between intervention condition and time (described above). Minoritized status did not play a role in any two-way interactions involving time or intervention condition. Moreover, Model 3 did not reveal a three-way interaction; however, the inclusion of the three-way interaction in the model revealed a previously suppressed two-way interaction between minoritized status and intervention condition.

**Figure 2:**
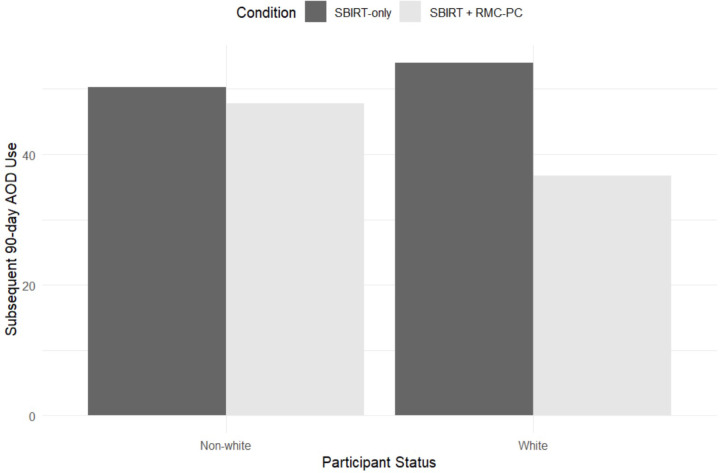
Predicted Values of AOD Use as a Function of Intervention Condition and Status as Racial Minoritized or White Participant *Note*. Expected values were computed for the first 90 days of the intervention and at mean levels of prior 90-day AOD use.

With the inclusion of the three-way interaction, the two-way interaction exists when time = 0 which represents the 90-day period beginning at baseline (i.e., study intake). Specifically, during the first 90 days of study participation, there were fewer days of AOD use for those in RMC-PC than SBIRT-only. However, this was a stronger effect for White participants than for participants with a minoritized status. Among White participants, the predicted value of subsequent 90-day AOD use was 53.97 for SBIRT-only versus 36.74 for RMC-PC; however, for participants with a minoritized status, the difference was only 50.32 for SBIRT-only and 47.75 for RMC-PC (see Fig. 2). Thus, during the first 90 days of the study, the effectiveness of RMC-PC versus the SBIRT-only control was minimal for those with a racial/ethnic minoritized status.

## DISCUSSION

There are reasons to believe SUD treatment may be less effective for returning citizens as well as for racial minoritized individuals [14]. Accordingly, we considered whether RMC-PC—an intervention designed to link people to treatment—would also be less effective for returning citizens and for those who identify as racial. Our analysis of data from a 12-month, randomized clinical trial of RMC-PC + SBIRT versus SBIRT-only supported this hypothesis. Specifically, multilevel analyses suggest RMC-PC is an effective intervention to reduce substance use compared to SBIRT-only, yet this effectiveness was stronger when participants had less time in jail immediately prior to study intake and for White, non-minoritized individuals.

In general terms, the effectiveness of the RMC-PC intervention is based on linking people to treatment and helping them initiate and become engaged in treatment [21, 29]. Indeed, Scott et al. (2023) found empirically that days of treatment mediated the association between RMC-PC versus control condition and days of abstinence [21]. In this study, the relative effectiveness of RMC-PC (compared to control) in reducing days of use was lower for those with more recent days in jail. That is, the effectiveness of RMC-PC appears to decline in the 90 days following release from jail, indicating a need for the intervention to be strengthened during this high-risk period. Returning citizens face many challenges that may have led to lower treatment utilization rates and, correspondingly, more days of use. For example, those recently incarcerated in jails may find difficulties with housing and employment, thus adding to daily living stressors that interfere with treatment engagement [36, 37]. There is also an overrepresentation of mental illness among those in jails [38], and comorbid mental health issues may interfere with the effectiveness of RMC-PC [27]. Moreover, the stressors associated with criminal-legal system involvement, such as the stigma of arrest and the disruption of social and economic stability, can significantly contribute to poorer health outcomes in general [39, 40]. Research has also shown returning citizens may not employ harm-reduction strategies as frequently as individuals who have not been incarcerated [14]. Given these challenges, programs that address the unique needs of returning citizens are clearly indicated.

Findings from this research align with previous literature investigating the intersection of race, substance use, and criminal-legal system involvement. Specifically, research has consistently shown that racial minorities, particularly those who identify as Black or Hispanic, are disproportionately affected by systemic biases within the criminal-legal system which leads to harsher sentencing and more limited access to treatment while incarcerated [41, 42]. Indeed, we found participants who identified as racial reported more days in jail prior to study enrollment than participants who identified as White. Of primary importance in this study, the effectiveness of RMC-PC relative to the SBIRT-only condition was stronger for White participants than for racial minoritized participants, underscoring the critical need for interventions that address the unique barriers faced by racial minorities. For instance, the strong link between substance use and criminal arrest may be particularly relevant to Black individuals, who are more likely to reside in disadvantaged neighborhoods where police presence is high and drug use is concentrated [43–45]. As such, these findings suggest that current treatment models like the RMC-PC may benefit from further adaptation to best meet the needs of high-risk and minority populations [39, 46, 47].

One potential tailored approach or adaptation of RMC to improve post-release outcomes for those who are incarcerated could be within the criminal-legal system. It may be useful to explore whether RMC can be implemented within carceral settings. In such a model, linkage managers could support linkage to treatment during incarceration and as part of release planning. Such a seamless continuity of care for returning citizens might have a greater impact, particularly for Black individuals who are disproportionally represented in this system.

In addition, and considering these results, it would be useful to explore expanding RMC’s traditionally narrow focus on treatment linkage and engagement to include education on harm reduction strategies for those who are not interested in or able to participate in SUD treatment. This could be particularly beneficial for those with incarceration histories who may not have had prior exposure to or lack knowledge of appropriate approaches to reduce substance use-related risks [14]. This would require providing RMC staff with training in both harm reduction approaches and the appropriate ways to integrate this information during motivational interviewing, a key element of the intervention [48].

Taken together, the moderation analyses suggest a key sub-group of patients at heightened vulnerability, namely individuals with recent incarceration history and those who are disproportionately of minority status. Although there is strong evidence of RMC-PC’s overall effectiveness, it is critical to identify ways to better help individuals at heighted risk for continued substance use and the ongoing risks of adverse health outcomes, including alcohol and drug-related morbidity and mortality.

### Limitations and Future Directions

These findings and implications should be considered in light of some limitations of this study. First, we had highly skewed data for jail time and for minoritized status. This might have compromised statistical power necessary to find effects among people who had longer incarceration histories prior to the study. In addition, the use of separate models to assess prior incarceration and minoritized status could potentially obscure the role of synergistic effects that exist between these two variables. Specifically, it is plausible that the interaction between being a racial minority and having an incarceration history generates unique barriers to SUD treatment that are not captured when these variables are examined separately. Due to the small sample size and high-level of skewness in these variables, however, this study is limited in its ability to explore these complex interactions fully, and thus, could even underestimate the true barriers to treatment faced by this subpopulation.

Second, there might have been value in examining multiple racial/ethnic identities beyond the minoritized identity of non-White. For example, examination of Black and Hispanic subgroups could be illuminating [17]. Addressing the intersecting roles of race/ethnicity and gender would also be important, especially focusing on the potential importance of masculinity on the health of returning citizens [49].

Finally, we focused on one outcome—days of substance use. Although this is a meaningful outcome, exclusion of other outcomes limits the generalization of the study to other important goals of RMC-PC, such as treatment engagement and retention. Ideally, a moderated mediation model could be tested that examines potential differences in the strength of indirect effects between intervention condition and days of use as a function of prior incarceration and racial, minoritized status. Adding additional potential moderators, such as experience of daily living stressors and/or psychiatric symptoms, would help build a model that could guide tailored interventions for those with SUD.

## CONCLUSIONS

RMC-PCs were developed to help reach individuals seen in FQHCs who might need SUD treatment [25]. Certainly, returning citizens and those holding a racial, minoritized status face a number of challenges that could impact linkage to and engagement with SUD treatment. We view this research study as an important exploratory step that would optimally lead to additional research focused on specific mechanisms that interfere with the effectiveness of interventions such as RMC-PC. In addition, this research suggests the potential benefit of possible adaptations of SUD interventions to meet the unique needs of these populations.

## Figures and Tables

**Table 1:** Treatment Days in the Past 90 Days by Intervention Condition and Wave

	RMC-PC + SBIRT condition		SBIRT-only condition	
Wave	*M*	*SD*	*M*	*SD*
3 months	13.99	26.37	7.59	22.76
6 months	14.14	28.77	8.19	24.23
9 months	13.42	29.03	8.20	24.00
12 months	9.98	25.90	5.73	20.50
Total	12.90	27.54	7.46	22.92

**Table 2:** Coefficients from Multilevel Regression for Jail Days Predicting Days of AOD Use

Predictor	Model 1	Model 2	Model 3
Prior AOD use	0.40***	0.39***	0.40***
Time	0.01	−0.46	−0.55
Condition	−3.57	−8.39**	−9.52**
Jail days	0.17	−0.08	−0.46
Time × condition	–	0.92*	1.18*
Time × jail days	–	0.00	0.09
Condition × jail days	–	0.57	1.45**
Time × condition × jail days	–	–	−0.20**

*Note.* Intervention condition was coded 1 = RMC-PC + SBIRT and 0 = SBIRT-only.

* *p* < .05.

** *p* < .01.

*** *p* < .001.

**Table 3:** Coefficients from Multilevel Regression for Minoritized Status Predicting Days of AOD Use

Predictor	Model 1	Model 2	Model 3
Prior AOD use	0.44***	0.44***	0.45***
Time	0.15	0.19	−0.71
Condition	−3.97	−22.03**	−31.05**
Minoritized status	3.06	−1.14	−5.68
Time × condition	–	0.94*	3.07
Time × minoritized status	–	−0.57	0.45
Condition × minoritized status	–	15.25	25.15*
Time × condition × minoritized status	–	–	−2.33

*Note.* Minoritized status was coded 1 = minoritized/racial and 0 = non-minoritized/White.

Intervention condition was coded 1 = RMC-PC + SBIRT and 0 = SBIRT-only.

* *p* < .05.

** *p* < .01.

*** *p* < .001.

## Data Availability

Due to the sensitivity of information related to substance use and treatment, the dataset is available only upon request and with proper data use agreements in place. All materials developed for the study are available upon request.

## Abbreviations

AOD: Alcohol or other drugs
AUDIT: Alcohol Use Disorder Identification Test
DAST: Drug Abuse Screening Test
FQHC: Federally Qualified Health Center
GAIN: Global Appraisal of Individual Needs
ICC: Intraclass correlation
RMC: Recovery management checkup
RMC-PC: Recovery management checkups done in primary care settings
SUD: Substance use disorder
SBIRT: Screening, Brief Intervention, and Referral to Treatment
